# The Fractional View of Complexity

**DOI:** 10.3390/e21121217

**Published:** 2019-12-13

**Authors:** António M. Lopes, J.A. Tenreiro Machado

**Affiliations:** 1 UISPA–LAETA/INEGI, Faculty of Engineering, University of Porto, R. Dr. Roberto Frias, 4200-465 Porto, Portugal; 2 Institute of Engineering, Polytechnic of Porto, Deptartment of Electrical Engineering, R. Dr. António Bernardino de Almeida, 431, 4249-015 Porto, Portugal; jtm@isep.ipp.pt

**Keywords:** fractals, fractional calculus, complex systems, dynamics, entropy, information theory

Fractal analysis and fractional differential equations have been proven as useful tools for describing the dynamics of complex phenomena characterized by long memory and spatial heterogeneity. There is a general agreement about the relation between the two perspectives, but the formal mathematical arguments supporting their relation are still being developed.

The fractional derivative of real order appears as the degree of structural heterogeneity between homogeneous and inhomogeneous domains. A purely real derivative order would imply a system with no characteristic scale, where a given property would hold regardless of the scale of the observations. However, in real-world systems, physical cut-offs may prevent the invariance spreading over all scales and, therefore, complex-order derivatives could yield more realistic models.

Information theory addresses the quantification and communication of information. Entropy and complexity are concepts that often emerge in relation to systems composed of many elements that interact with each other, which appear intrinsically difficult to model.

This Special Issue focuses on the synergies of fractals or fractional calculus and information theory tools, such as entropy, when modeling complex phenomena in engineering, physics, life, and social sciences. It includes 16 manuscripts addressing novel issues and specific topics that illustrate the role of entropy-based techniques in fractality, fractionality, and complexity. In the follow-up the selected manuscripts are presented in alphabetic order.

The manuscript “A Fractional-Order Partially Non-Linear Model of a Laboratory Prototype of Hydraulic Canal System”, by Saddam Gharab, Vicente Feliu-Batlle and Raul Rivas-Perez, addresses the identification of the nonlinear dynamics of the main pool of a laboratory hydraulic canal installed in the University of Castilla La Mancha. A new dynamic model is developed by taking into account the measurement errors caused by the different parts of the experimental setup. Fractional and integer order plus time delay models are used to approximate the responses of the main pool of the canal in its different flow regimes [1].

In the paper “Adaptive Synchronization Strategy between Two Autonomous Dissipative Chaotic Systems Using Fractional-Order Mittag-Leffler Stability”, Licai Liu, Chuanhong Du, Xiefu Zhang, Jian Li and Shuaishuai Shi investigate two novel four-dimensional, continuous, fractional-order, autonomous, and dissipative chaotic system models with high complexity. Based on the fractional Mittag-Leffler stability theory, an adaptive, large-scale, and asymptotic synchronization control method is derived [2].

In “An Entropy Formulation Based on the Generalized Liouville Fractional Derivative”, Rui A. C. Ferreira and J. Tenreiro Machado present a new entropy formula based in the Liouville fractional derivative. The new concept is illustrated when applied to the Dow Jones Industrial Average time series. The Jensen–Shannon divergence is also generalized and its variation with the fractional order is tested [3].

In the work “Analytical Solutions of Fractional-Order Diffusion Equations by Natural Transform Decomposition Method”, Rasool Shah, Hassan Khan, Saima Mustafa, Poom Kumam and Muhammad Arif, solve fractional-order diffusion equations using the natural transform decomposition method. Numerical examples are presented, showing that the procedure involves a small volume of calculations and has a high rate of convergence compared to other analytical techniques [4].

The paper “Application of the Laplace–Adomian Decomposition Method for the Analytical Solution of Third-Order Dispersive Fractional Partial Differential Equations”, authored by Rasool Shah, Hassan Khan, Muhammad Arif and Poom Kumam, investigates the analytical solution of fractional-order dispersive partial differential equations using the Laplace–Adomian decomposition method. The effectiveness and convergence rate is illustrated with examples, confirming the validity of the method [5].

In the manuscript “Approximation to the Hadamard Derivative via the Finite Part Integral”, Chuntao Yin, Changpin Li and Qinsheng Bi present rectangular and trapezoidal formulas to approximate the Hadamard derivative, following the idea of the finite part integral. They apply the proposed numerical method to the differential equation with the Hadamard derivative. Several numerical examples are presented to show the effectiveness of the technique [6].

The paper “Complexity Analysis of Escher’s Art”, by António M. Lopes and J.A. Tenreiro Machado, adopts complexity indices, dimensionality-reduction and visualization techniques for studying the evolution of Escher’s art. Grayscale versions of 457 artworks are analyzed by means of complexity indices and represented using the multidimensional scaling technique. The results are correlated with the distinct periods of Escher’s artistic production. The time evolution of the complexity and the emergent patterns demonstrate the effectiveness of the approach for a quantitative characterization of art [7].

The work “Descriptions of Entropy with Fractal Dynamics and Their Applications to the Flow Pressure of Centrifugal Compressor”, authored by Yan Liu, Dongxiao Ding, Kai Ma and Kuan Gao, introduces and develops entropy related topics to describe the fractal dynamics of flow pressure and its intrinsic properties. The authors study the relationships between fractal dynamics and the real time series of outlet flow pressure for a centrifugal compressor. Their results confirm the feasibility of the method to identify a surge accurately [8].

In the research paper “Entropy Analysis of Soccer Dynamics”, António M. Lopes and J.A. Tenreiro Machado adopt the information and fractional calculus tools for studying the dynamics of a national soccer league. A soccer league season is treated as a complex system with a state observable at discrete time instants, and is processed by means of different tools. The complex system behavior is visualized in 3-D maps generated by multidimensional scaling, which allows a direct interpretation of the results [9].

The manuscript “Entropy Production Rates of the Multi-Dimensional Fractional Diffusion Processes”, by Yuri Luchko, derives and analyzes the fundamental solution to the *n*-dimensional time-space-fractional partial differential equation with the Caputo time-fractional derivative of order *b*, 0<b<2, and the fractional spatial derivative (fractional Laplacian) of order *a*, 0<a≤2. An explicit formula for the entropy production rate of the fractional diffusion processes is presented [10].

In the paper “Fractional Refined Composite Multiscale Fuzzy Entropy of International Stock Indices”, Zhiyong Wu and Wei Zhang propose the fractional refined composite multiscale fuzzy entropy to quantify the complexity dynamics of international stock indices. They show that the proposed entropy-based measure outperforms the traditional indices [11].

The research work “Memories of the Future. Predictable and Unpredictable Information in Fractional Flipping of a Biased Coin”, by Dimitri Volchenkov, reports the exact amounts of predictable and unpredictable information in flipping a biased coin. These information components vary smoothly with the fractional order parameter. The destructive interference between two incompatible hypotheses about the flipping outcome culminates in a fair coin, which stays fair also for fractional flipping [12].

The manuscript “Model Order Reduction: A Comparison between Integer and Non-Integer Order Systems Approaches”, by Riccardo Caponetto, José Tenreiro Machado, Emanuele Murgano and Maria Gabriella Xibilia, compares classical and non-integer model order reduction methodologies. An open loop balanced realization is compared with three approaches based on a non-integer representation of the reduced system. Case studies are considered and the results confirm the capability of fractional order systems to capture and compress the dynamics of high order systems [13].

In the research “New Texture Descriptor Based on Modified Fractional Entropy for Digital Image Splicing Forgery Detection”, by Hamid A. Jalab, Thamarai Subramaniam, Rabha W. Ibrahim, Hasan Kahtan and Nurul F. Mohd Noor proposes a new scheme for improving the accuracy of image splicing detection with low-dimension feature vectors. The methodology is based on the approximated Machado fractional entropy of the discrete wavelet transform. A superior detection accuracy and positive and false positive rates were achieved, compared with other state-of-the-art approaches, with low-dimension feature vectors [14].

The work “On the Complexity Analysis and Visualization of Musical Information”, by António M. Lopes and J.A. Tenreiro Machado, adopts several distinct mathematical and computational tools, namely complexity, dimensionality-reduction, clustering, and visualization techniques, for characterizing music. Digital representations of musical works of four artists are analyzed by means of distinct indices and visualized using the multidimensional scaling technique. The results are then correlated with the artists’ musical production. The patterns found in the data demonstrate the effectiveness of the approach for assessing the complexity of musical information [15].

In the manuscript “Parallel Implementation of Modeling of Fractional-Order State-Space Systems Using the Fixed-Step Euler Method”, Rafał Stanisławski and Kamil Kozioł present new results in the implementation of parallel computing for modeling fractional-order state-space systems. Two different parallelization approaches are proposed. Simulation results illustrate the high efficiency of the introduced schemes [16].

The guest editors believe that the selected high-quality papers will help scholars and researchers to push forward the progress in the emerging area of Complex and Fractional Dynamical systems.
